# Embodied Intelligence for Soil Health toward Sustainable Smart Agriculture: Sensing the Soil with Flexible Geotextiles Sensors

**DOI:** 10.1002/gch2.70117

**Published:** 2026-06-08

**Authors:** Ayse Feyza Yilmaz, Meltem Tekcin, Burak Kilic, Erdem Bahadir, Fatima Iqbal, Ahad Khabbaz Bavil, Batuhan Atasoy, Senem Kursun

**Affiliations:** ^1^ Faculty of Mechanical Engineering Mechanical Engineering Department Istanbul Technical University Istanbul Türkiye; ^2^ Faculty of Textile Technologies and Design Textile Engineering Department Istanbul Technical University Istanbul Türkiye; ^3^ Faculty of Engineering and Natural Sciences Mechanical Engineering Department Işık University Istanbul Türkiye; ^4^ Faculty of Mechanical Engineering System Dynamics and Control Department Istanbul Technical University Istanbul Türkiye; ^5^ Faculty of Computer and Informatics Engineering Computer Sciences Department Istanbul Technical University Istanbul Türkiye; ^6^ Faculty of Mechanical Engineering Mechatronics Engineering Department Istanbul Technical University Istanbul Türkiye; ^7^ CETEX Center of Excellence for Textiles ITU ARI TEKNOKENT Istanbul Türkiye

**Keywords:** artificial intelligence (AI), flexible soil sensors, geotextiles, IoT, smart and precision agriculture, soil health, sustainability, textile‐based sensors

## Abstract

The integration of agriculture and Internet of Things (IoT) technology has transformed traditional farming into data‐driven, automated, and highly interactive systems. Central to this evolution are sensor platforms that monitor soil conditions and environmental parameters such as moisture, temperature, pH, nutrients, and pollutants. Recent progress in flexible electronics and smart functional materials has created new opportunities for developing adaptable sensor systems suited to dynamic agricultural applications. Despite these advancements, the use of geotextiles as soil‐sensing platforms remains largely unexplored. This review traces the shift from conventional rigid soil sensors to flexible and potentially geotextile‐based systems, assessing their roles in IoT‐enabled agricultural applications. While soil sensors are generally discussed under separate headings, the review integrates them under the common themes, including material selection, fabrication methods, testing methodologies, energy management strategies, and IoT connectivity. By identifying common materials, design principles, and manufacturing techniques across various soil sensors, the review proposes a synthesis framework that supports multi‐sensor integration. Extending this integrative approach to geotextile‐based structures envisions the realization of flexible, large‐area, and adaptive soil sensing platforms that could enable next‐generation of sustainable and smart precision agriculture.

## Graphical abstract

This review examines the transition from rigid to flexible soil sensing technologies in the context of soil as a dynamic and heterogeneous environment. By integrating sensor types, sensing modalities, materials, fabrication and deployment strategies, and IoT‐enabled energy and communication approaches, it highlights geotextiles as scalable sensing platforms and discusses key challenges and future directions for sustainable and resilient agricultural systems.

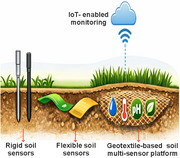

## 1 Introduction

Despite the transformative influence of artificial intelligence, digitalization, and data‐driven economies, agriculture remains the indispensable foundation of human survival and societal stability [1, 2, 3, 4, 5]. Although short‐term subsistence could be achieved through hunting, gathering, and fishing, such practices inherently exceed the regenerative capacity of natural systems, ultimately leading to resource depletion, food insecurity, migration, and ecological collapse [6, 7, 8, 9]. In contrast, agricultural production is fundamentally sustained by the chemical, physical, and biological fertility of soils, which regulate nutrient availability, water retention, and ecosystem functionality; thus, well‐managed agricultural systems not only ensure food security but also preserve essential processes such as the carbon and water cycles and maintain biodiversity [10, 11]. However, rapid population growth, increasing urbanization, diminishing water resources [12, 13], and the escalating impacts of climate change are placing unprecedented pressure on agricultural systems to enhance productivity while ensuring sustainability [14, 15, 16, 17]. Traditional farming methods, largely reliant on experience and manual observation, often result in inefficient resource use, declining production quality, and environmental degradation, challenges that are further intensified by stressors such as drought, salinity, erosion, and biotic factors [18, 19, 20, 21, 22]. Consequently, the integration of data‐driven approaches has become a critical necessity in modern agriculture [23, 24, 25], particularly through the adoption of IoT technologies, which enable real‐time monitoring, automated decision‐making, and remote management via interconnected sensors, communication networks, and cloud‐based platforms, thereby facilitating optimized resource utilization, increased productivity, and reduced environmental impact [26, 27].

The effective implementation of precision agriculture and smart farming applications depends on the development of soil sensor technologies [28, 29, 30]. The foundation of agricultural sensors was laid with the development of the first electrical devices designed to measure soil moisture and temperature; it has since expanded to include systems that measure parameters such as pH, conductivity, gas composition, nutrient content, and microbial activity [31, 32, 33]. Although methods such as gravimetric (oven drying) that provide high accuracy are available for measuring soil parameters, such as soil moisture, sensors offer significant advantages such as the ability to perform continuous measurements in the field, obtain real‐time data, monitor spatial heterogeneity across large areas, and support agricultural management decisions through automatic data recording [34, 35, 36, 37]. However, the performance of soil sensors is strongly determined by the quality of physical contact with the soil, the material and structural properties of the sensor, and its environmental robustness [32, 33, 34, 35, 36, 37, 38].

In recent years, advances in flexible electronics and smart material technologies have brought about a shift from rigid (solid) sensors to flexible sensors [39]. Air gaps and microcracks that form around the sensor in the soil can significantly reduce measurement accuracy and limit the sensor's response to environmental changes. This problem weakens data reliability, especially in long‐term field measurements, and reduces the effectiveness of real‐time monitoring systems [37, 38, 39, 40, 41]. Flexible sensors, thanks to their robust structures resistant to mechanical deformations such as bending, twisting, and folding, adapt to the natural texture of the soil, minimizing air gaps; thus, significantly improving measurement accuracy, signal stability, and field durability [39, 40, 41, 42]. Due to their low cost, lightweight, and multi‐surface compatibility, flexible sensors are now regarded as pioneers of a new technological era in agricultural monitoring systems. However, real‐time in‐soil sensors are still immature; therefore, the development of low‐cost, reliable, and non‐invasive sensors remains a critical priority for advancing precision agriculture.

While flexible film sensors effectively address geometric incompatibilities at the soil‐sensor interface (conformability), their non‐porous and compact structure disrupts ion mobility, gas diffusion, thermal transfer, and hydraulic continuity, thereby generating an artificial micro‐environment around the sensor that is decoupled from the true dynamics of the soil and ultimately constrains data reliability. To overcome this technical bottleneck, geotextiles, a largely unexplored research domain in agricultural monitoring systems, represent the next step in flexible soil electronics.

Geotextiles, which are widely used in geotechnical applications such as soil stabilization, erosion control and drainage, enable sensor integration without interfering with the soil's natural hydraulic, thermal and gas transfer dynamics, thanks to their woven, non‐woven or knitted polymeric fiber structures [43]. In an agricultural context, these materials not only mitigate deep percolation losses of irrigation water, thereby enhancing water use efficiency in the root zone, but also leverage their intrinsic porosity to facilitate the efficient capillary transport of liquids and gases directly to the sensing elements. By directly integrating conductive materials into these matrices, geotextiles are transformed from a passive substrate into an active transducer. This technological synergy creates a high‐precision, scalable ‘smart textile’ platform capable of simultaneously converting critical parameters such as moisture, temperature, pH and nutrient content into digital data, without disrupting the soil's natural flow and respiration process (air permeability).

**FIGURE 1:**
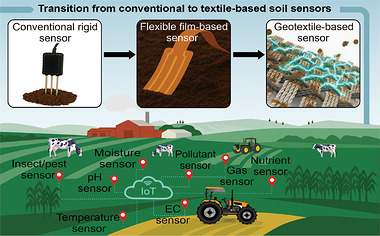
Schematic illustration of soil IoT sensor technologies in natural environments. Overview of the transition from conventional rigid soil sensors to flexible film‐based and geotextile‐based sensing platforms in smart agriculture, where integrated soil sensors are interconnected through IoT networks.

This study provides an overview of current soil sensor technologies and aims to establish a structured framework for the development of geotextile‐based soil sensing platforms (Figure 1). While the literature predominantly classifies sensors according to individual measurands such as moisture, temperature, pH, pollutants, nutrient content, conductivity, and gases, this review adopts a system‐level organization that follows the evolution of soil sensing technologies. It begins with soil as a dynamic measurement environment and progresses through the transition from rigid to flexible sensor architectures and their deployment in soil matrices. Soil sensor types and operating mechanisms are then discussed in relation to sensing modalities and signal transduction principles. A dedicated section focuses on material systems, including substrates, conductive components, and functional materials, which collectively govern sensor performance and environmental stability. Building on this foundation, geotextiles are introduced as emerging substrates for permeable and mechanically compliant soil sensing systems. The review further examines fabrication strategies and scalable manufacturing approaches for flexible sensor architectures. Subsequently, soil parameter measurement and data analysis approaches are discussed, highlighting signal interpretation, calibration, and system‐level performance evaluation. Finally, energy management strategies, wireless communication technologies, and IoT integration are addressed as essential components for autonomous, real‐time, and distributed soil sensing networks. By integrating these interconnected layers, the review establishes a transferable design framework for the development of scalable, multimodal, and sustainable geotextile‐based sensing systems.

## 2 Soil as Dynamic Measurement Environment

Soil is a complex system of nature formed by the interaction of living and non‐living components and generally consists of four main components: mineral matter, organic matter, water, and air [44]. Mineral matter constitutes the bulk of the soil, forming its structural skeleton; including sand, silt, clay, as well as calcium carbonate (CaCO_3_), iron oxides (Fe_2_O_3_), aluminum oxides (Al_2_O_3_), feldspars, quartz (SiO_2_), and clay minerals (e.g., montmorillonite, illite, kaolinite) [45, 46, 47]. Organic matter is formed by the decomposition of plant and animal remains and is found in the form of humus, which increases soil fertility [48]. Soil water contains dissolved ions (e.g., nitrate (NO_3_^−^), ammonium (NH_4_^+^), potassium (K^+^), calcium (Ca^2+^), magnesium (Mg^2+^), sulfate (SO_4_^2+^), and phosphate (PO_4_^3−^)) and provides nutrient transport for plants [49]. Soil air comprises gases such as oxygen (O_2_), carbon dioxide (CO_2_), and nitrogen (N_2_), and is necessary for root respiration and microbial activity [50, 51]. The continuity of the physicochemical and biological interactions between these components gives the soil a heterogeneous and dynamic system character.

The relative proportions of soil components vary depending on factors such as climate, parent material, vegetation, topography, and time [52]. This variability occurs both in the overall composition and across different spatial scales, leading to the formation of horizontal and vertical heterogeneity in soil properties As an example, in a given agricultural region, clay content in the surface layer (0–20 cm) has been reported to range from 371.2 to 432.0 g/kg, with available phosphorus (P) concentrations spanning from 221.34 to 418.63 mg/kg. Vertically, nutrient concentrations often decrease sharply with depth; for example, P and K^+^ levels can drop from 418.63 and 261.43 mg/kg in the top 20 cm to 258.99 and 164.78 mg/kg, respectively, just 20 cm deeper (20–40 cm sub‐surface layer) which highlights the strong spatial variability [53]. These variations in composition and spatial distribution directly influence the chemical and biological functions of the soil [54, 55].

This spatial and compositional variability is further reflected in the distribution of biological activity within the soil. Soil hosts a diverse community of microorganisms responsible for decomposing organic matter and transforming nutrients into plant‐available forms [56, 57]. However, this biological activity is not uniformly distributed throughout the soil but is particularly concentrated in the rhizosphere, the dynamic and narrow zone surrounding plant roots [58]. In this region, intricate interactions between roots, microorganisms, and soil components regulate nutrient availability at a micro‐scale [59]. Furthermore, soil continuously interacts with the atmosphere through gas emissions (e.g., CO_2_, methane (CH_4_) and nitrous oxide (N_2_O)) and wind erosion, which further contribute to its highly dynamic and heterogeneous nature.

Due to this high degree of spatial and temporal heterogeneity, conventional bulky monitoring systems face significant challenges because they lack the spatial resolution required to capture localized gradients and often provide only an averaged, less representative view of the soil's true chemical state. Furthermore, the inherent nature of this environment, which is characterized by continuous fluctuations in moisture, chemical flux, and microbial growth, promotes degradation mechanisms such as corrosion, biofouling, and mechanical wear. Consequently, bridging the gap between micro‐scale biological processes and robust in situ measurement remains a major challenge for ensuring the long‐term accuracy and reliability of soil sensors.

## 3 Transition from Rigid to Flexible Soil Sensors

Soil sensing technologies are generally categorized into rigid and flexible platforms depending on substrate mechanics. Rigid sensors are developed on mechanically non‐deformable substrates that maintain a fixed geometry during operation [60]. They are generally preferred in applications requiring stable form factors and high structural durability. They have been developed using microelectronics and industrial fabrication techniques such as printed circuit board (PCB) manufacturing, photolithography, and silicon‐based processing [61], which provide high reproducibility but limit adaptability in heterogeneous soil environments.

The gypsum block system, introduced in the United States in the 1940s, represents among the earlies rigid sensing technologies capable of providing quantitative estimates of soil moisture. These sensors consist of two metal electrodes embedded within a porous gypsum matrix, where changes in soil water content alter the electrical conductivity between electrodes, enabling indirect moisture estimation through resistance measurements [62]. By the late 1960s and 1970s, rigid sensing approaches expanded beyond moisture measurement, as soil temperature (thermistors), electrical conductivity (EC) sensors, and pH (glass electrodes) were increasingly quantified using electrical response‐based principles [31, 63, 64].

During the following decades, several rigid soil moisture sensing technologies became widely adopted, including tensiometers, capacitance/frequency domain reflectometry (FDR) probes, and time domain reflectometry (TDR) sensors. Among these, TDR systems, based on electromagnetic wave propagation and soil dielectric permittivity, emerged as a benchmark technique for high‐accuracy volumetric water content estimation in both laboratory and field conditions.

Despite their widespread usage, rigid soil sensors exhibit inherent limitations in mechanically dynamic and heterogeneous soil environments. Soil is a non‐uniform, multiphase medium that undergoes swelling, shrinkage, cracking, and compaction, and contains heterogeneous inclusions such as stones, pores, and organic matter. Under these conditions, rigid probes often experience imperfect soil sensor contact, resulting in air gaps that distort dielectric or resistive measurements and lead to systematic underestimation of soil moisture content [42, 65].

Experimental studies have shown that installation‐induced soil disturbance can introduce measurable errors in rigid soil moisture sensing systems, particularly TDR‐based probes. Depending on soil texture and installation method, deviations in volumetric water content on the order of approximately 2–6% have been reported, primarily due to changes in bulk density, probe–soil contact quality, and air‐gap formation around the rods. These effects are especially pronounced in fine‐textured and structured soils, where small variations in compaction substantially alter the effective dielectric permittivity sensed by the probe. Overall, these findings indicate that rigid sensor performance is highly dependent on installation quality and site‐specific calibration conditions [66]. For conductivity and electrode‐based systems, additional limitations arise from corrosion and polarization effects in ion‐rich soils, which can contribute to signal drift and long‐term instability. Collectively, these constraints, along with the inability of rigid sensors to conform to irregular root structures and evolving soil geometries, have motivated the development of mechanically compliant sensing systems capable of maintaining stable and conformal soil–sensor interfaces.

In the early 2000s, advances in materials science and micro/nanoelectronics enabled the emergence of a new generation of agricultural sensing platforms based on flexible substrates and functional nanomaterials. Flexible sensors are typically fabricated on mechanically compliant polymer substrates such as polydimethylsiloxane (PDMS), polyethylene terephthalate (PET), and polyimide (PI). These materials gained prominence due to their distinct advantages for soil sensing applications: PDMS offers high elasticity and stretchability, making it suitable for deformation‐prone environments; PET provides low‐cost processing and compatibility with scalable roll‐to‐roll manufacturing; and PI exhibits superior thermal and chemical stability, supporting long‐term outdoor deployment under harsh environmental conditions [67, 68, 69, 70]. Compared with rigid substrates, these polymeric platforms can bend and conform to irregular soil structures, thereby improving soil–sensor coupling and minimizing air‐gap‐induced measurement artifacts.

In parallel, next‐generation conductive materials including carbon nanotubes (CNTs), graphene, and conductive polymers such as poly(3,4‐ethylenedioxythiophene):poly(styrenesulfonate) (PEDOT:PSS) and polyaniline (PANI) were increasingly integrated as sensing layers and electrodes. These materials were adopted because they combine high electrical conductivity with mechanical flexibility, enabling stable sensing performance under bending, stretching, and repeated deformation [67, 68, 69, 70]. CNTs and graphene provide high conductivity and large effective surface area, supporting highly sensitive resistive and capacitive sensing mechanisms, although their performance may be influenced by dispersion quality, percolation stability, and environmental degradation. Conductive polymers such as PEDOT:PSS and PANI exhibit mixed ionic–electronic conduction, which is particularly advantageous in soil environments where moisture dynamics are strongly coupled to ion transport. Nevertheless, polymer‐based conductors may undergo aging, swelling, or conductivity drift under prolonged exposure to water, salinity, and temperature cycling, often necessitating encapsulation strategies or material optimization for reliable field deployment [71].

Overall, the integration of flexible substrates with functional conductive materials established a design paradigm in which sensors could preserve electrical functionality while mechanically adapting to soil deformation and complex biological interfaces. Although flexible sensors were initially developed for biomedical applications, such as wearable and skin‐mounted devices, their relevance to agricultural monitoring was rapidly recognized due to the similarly complex and mechanically active nature of plant–soil systems. Early studies published around 2008 reported some of the first polymer‐based flexible soil moisture sensor prototypes, demonstrating the feasibility of conformal sensing architectures and establishing the technological foundation for modern flexible agricultural sensor platforms [72, 73].

## 4 Deployment of Flexible Sensors in Soil

Flexible sensors are typically deployed below the soil surface using three main strategies: (i) horizontal placement, (ii) vertical insertion, and (iii) integration with cylindrical support structures such as pipes or probe rods. Unlike rigid sensors, flexible sensors establish more uniform contact across the sensing interface [39, 40, 41, 42]. Among these deployment modes, horizontal installation at a specific depth is particularly effective, as it enables wide‐area monitoring of moisture, pH, and ionic distribution over the rhizosphere. The coplanar plate capacitor (CPC) architecture on PET film substrates provides a narrow vertical sensing footprint suitable for depth‐resolved horizontal profiling, outperforming interdigital capacitor (IDC) designs for this application [74]. Field evaluations have demonstrated that horizontal sensor networks detected spatial moisture differences exceeding 0.05 m^3^·m^−^^3^ within a single greenhouse, confirming the feasibility of area‐based monitoring with flexible film sensors [75]. Given that soil properties vary significantly even at the meter scale due to differences in soil type, compaction, and management history, distributed horizontal arrays are especially relevant for resolving local heterogeneity in the rhizosphere [52, 53].

Vertical placement of flexible sensors enables multi‐layered data acquisition at different soil depths, allowing the extraction of depth‐based profiles of nutrient and water dynamics inside the root zone. A low‐cost soil moisture profile probe, comprising copper (Cu) capacitors printed on PET film and rolled onto a poly(vinyl chloride) (PVC) pipe for vertical insertion, has been developed to measure volumetric water content (VWC) simultaneously at 10, 20, and 30 cm depths. Field trials in grape vineyards and greenhouses confirmed that the sensor captured dynamic VWC changes in response to precipitation and irrigation. However, a stabilization period of 10 to 14 days was required after insertion to allow the disturbed soil to consolidate around the film capacitors, during which readings drifted by 0.02 to 0.07 m^3^·m^−^^3^ [75]. An accidental physical disturbance during field maintenance resulted in an immediate decrease in VWC at all depths, highlighting the importance of maintaining stable soil–sensor contact. These observations underscore the need for minimally invasive installation protocols and deployment geometries that limit soil disturbance during insertion [75, 76].

A third deployment strategy integrates sensing elements with cylindrical support structures, such as pipes or probe rods, inserted into the soil. In this configuration, the pipe or rod works as both a mechanical carrier and an access pathway for spatially distributed measurements. A mobile sensor platform capable of traversing subsurface PVC pipeline networks buried at a depth of 0.2 m has been developed for real‐time soil moisture monitoring, enabling the construction of two‐ and three‐dimensional moisture maps at meter‐scale resolution; a pipe spacing of 0.6 m was sufficient to capture micro‐variability in soil water content [77]. Similarly, coil‐based moisture sensors mounted onto PVC pipe supports have been deployed in wireless networks for precision irrigation, allowing moisture measurements at multiple depths along the pipe axis [78]. Such pipe‐supported configurations offer advantageous in agricultural settings where buried or probe‐like supports provide mechanical stability and facilitate distributed monitoring. The conformability of flexible substrates is designed to improve such systems by enabling conformal wrapping around pipes or rods to improve soil–sensor contact along curved interfaces, although this ability remains to be experimentally validated under field conditions. Although these advancements exist, challenges persist across all deployment strategies, including sensor drift due to biological fouling and organic matter accumulation [76, 79], as well as the limited number of prototypes validated under multi‐season field conditions [76, 80, 81, 82].

## 5 Soil Sensor Types and Operating Mechanisms

Soil sensors used in agricultural applications are classified according to the parameters they measure, including soil moisture, temperature, pH, nutrients, EC, gases, biological activity (insects/pests), and pollutants [52].

Soil moisture is a fundamental parameter for evaluating soil health and ensuring optimal plant growth, as it serves as the primary water source for physiological activities. It significantly modulates the physicochemical properties of soil, thereby affecting nutrient dissolution, ion uptake, and microbial dynamics. To accurately measure volumetric water content by exploiting changes in the dielectric or electrical properties of soil, soil moisture sensors are used. The main sensing approaches includes TDR and FDR, which estimate moisture content based on the dielectric permittivity of soil; capacitive sensors, which measure changes in capacitance due to water's high dielectric constant; and resistive sensors, which rely on increased ionic conductivity in wetter soils [83, 84]. Additionally, neutron probe methods are used in research‐grade applications. These techniques differ significantly in penetration depth, sensitivity to soil salinity, and compatibility with flexible substrates. Capacitive and resistive approaches are generally more suitable for low‐cost and flexible implementations, whereas TDR/FDR systems offer higher accuracy but require more complex electronics [85, 86].

*Soil pH sensors* determine soil acidity (pH < 7) or alkalinity (pH > 7) by measuring hydrogen ion (H^+^) activity at the sensor interface. Conventional glass electrode pH sensors operate based on potentiometric measurements using a hydrogen ion‐selective glass membrane in combination with a reference electrode, following the Nernstian relationship between membrane potential and ion activity [87]. Emerging alternatives include ion‐sensitive field‐effect transistors (ISFETs), which convert ion activity into variations in surface potential at a semiconductor gate interface, and metal‐oxide‐based sensing layers used in in potentiometric or field‐effect configurations. ISFETs are particularly attractive for miniaturized and integrated systems due to their compatibility with integrated circuit design; however, they require careful encapsulation to operate reliably in complex soil environments [88, 89]. Soil pH critically affects nutrient bioavailability and plant physiology; thus, accurate and stable sensing across varying soil conditions is essential [58, 90].

Soil temperature is another vital parameter, as it directly influences key agricultural processes such as germination, blooming, and composting, while also governing the physical, chemical, and microbiological activities essential for plant growth. Its behavior is complex, being strongly dictated by soil properties like thermal conductivity, water content, and surface covering materials [91]. *Soil temperature sensors* measure soil temperature using devices whose electrical properties vary predictably with temperature. Common implementations include thermistors (temperature‐dependent resistors with high sensitivity) (typically negative temperature coefficient (NTC) thermistors), resistance temperature detectors (RTDs) (typically metal‐based sensors with high accuracy and stability), and thermocouples, which generate a voltage based on temperature gradients between junctions (Seebeck effect). While thermistors are widely used in low‐cost soil monitoring systems, RTDs and thermocouples are preferred in applications requiring higher precision and wider temperature ranges [92]. For flexible systems, printed resistive temperature sensors are gaining attention due to their mechanical compliance and integration capability [93].

*Soil nutrient sensors* are designed to monitor essential macronutrients in the form of dissolved ionic species, such as NO_3_^−^, K^+^, and PO_4_^3^^−^, which are fundamental to plant growth and metabolic processes [94]. However, maintaining optimal nutrient levels is challenging, as both deficiency and excess can adversely affect crop productivity and lead to environmental issues such as eutrophication and ecosystem imbalance. Conventional soil nutrient analysis is typically performed by collecting soil samples and analyzing them in centralized laboratories using techniques such as atomic absorption spectroscopy (AAS), ultraviolet‐visible (UV‐vis) spectroscopy, and gas chromatography‐mass spectrometry (GC‐MS) [95, 96]. While these methods provide high analytical accuracy, they are labor‐intensive, time‐consuming, and require skilled personnel, making them unsuitable for real‐time and in situ monitoring. To overcome these limitations, in situ sensing platforms have emerged as a practical alternative, enabling real‐time or near‐continuous monitoring directly within the soil environment [97, 98]. Among these, electrochemical sensing approaches provide direct and ion‐specific detection with low power consumption by transducing ionic activity into measurable electrical potentials or currents. In particular, ion‐selective electrodes (ISEs) are considered one of the most mature technologies for soil applications [99, 100, 101, 102, 103]. In contrast, EC‐based sensors provide only indirect and non‐selective information about overall ionic content. However, long‐term deployment of such systems remains challenging due to issues such as signal drift, electrode fouling, and mechanical instability in heterogeneous soil environments. Recent advances have focused on improving sensor robustness and stability through solid‐contact architectures and the use of conducting polymers and carbon‐based nanomaterials, as well as the development of flexible and printed sensor platforms for reliable long‐term in situ deployment under dynamic soil conditions.

*Soil gas sensors*, on the other hand, detect gaseous compounds such as CO_2_, CH_4_, ammonia (NH_3_), nitrogen oxides (NO_x_) and N_2_O emitted from soil. These sensors operate using electrochemical cells (based on redox reactions), non‐dispersive infrared (NDIR) optical sensing (for gases like CO_2_), or metal‐oxide semiconductor (MOS) sensors that detect gas‐induced resistance changes. Each method differs in selectivity, power consumption, and environmental sensitivity. Integration into flexible systems remains challenging, particularly for optical and high‐temperature MOS sensors, but advances in nanomaterials are enabling new possibilities [104].

*Soil EC* sensors measure the ability of soil to conduct electrical current, which reflects ion concentration and salinity. Two main approaches exist: contact (electrode‐based) methods, where current is injected directly into the soil via electrodes, and non‐contact methods, such as inductive or electromagnetic sensors, which infer conductivity without direct electrical contact. While contact sensors are simpler and widely used, they are prone to electrode polarization and degradation. Non‐contact methods are more robust but require more complex circuitry, making them less suitable for low‐cost flexible implementations [105, 106, 107].

*Soil insect/pest sensors* detect biological activity using acoustic, vibrational, or biochemical sensing mechanisms. Acoustic and vibration‐based sensors identify characteristic movement patterns or feeding sounds of soil organisms, while biochemical sensors detect specific biomarkers. These systems are still emerging and often rely on signal processing and machine learning for accurate classification [108].

*Soil pollutant sensors* monitor contaminants such as heavy metals (lead (Pb^2^^+^) cadmium (Cd^2^^+^) and mercury (Hg^2^^+^)), pesticides, and organic pollutants using electrochemical (e.g., voltammetry, amperometry), optical (e.g., spectroscopy), or biosensing techniques. Electrochemical methods are particularly suitable for trace detection due to their high sensitivity and compatibility with miniaturized and printed sensor platforms [109].

**FIGURE 2:**
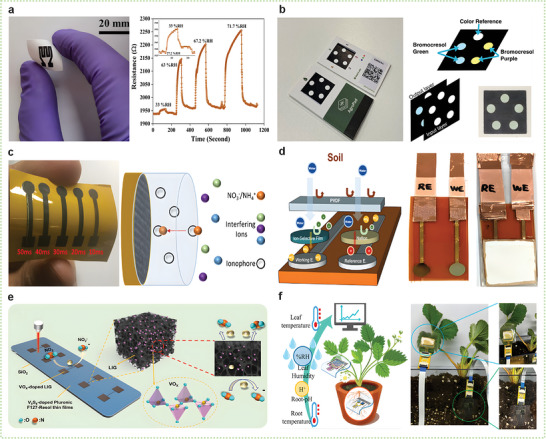
Representative flexible soil sensors reported in literature. (a) Flexible paper‐based flexible humidity sensor using screen‐printed graphene–carbon ink. Reproduced under terms of the CC‐BY license [144]. Copyright 2023, published by Elsevier. (b) Colorimetric paper pH sensor integrated with a smartphone‐based machine learning system for rapid on‐site soil analysis. Reproduced under terms of the CC‐BY license [149]. Copyright 2025, published by PLOS. (c) Laser‐induced graphene (LIG) ion‐selective sensor for detecting NH_4_^+^ and NO_3_^−^ ions in soil samples. Reproduced with permission [80]. Copyright 2018, published by ACS. (d) Inkjet‐printed NO_3_^−^ sensor with polyvinylidene fluoride (PVDF) protective layer enabling direct sensing in moist soil environments. Reproduced under terms of the CC‐BY license [152]. Copyright 2024, published by Wiley. (e)Vanadium oxide (VO_x_)‐doped LIG multimodal sensor capable of decoupled detection of NO_x_ and temperature for precision agriculture. Reproduced under terms of the CC‐BY license [163]. Copyright 2023, published by Wiley. (f) Lead‐free perovskite/poly(vinylidene fluoride‐co‐hexafluoropropylene) (PVDF‐HFP) composite multimodal sensor for simultaneous pH and humidity monitoring in crop environments. Reproduced with permission [164]. Copyright 2024, published by American Chemical Society.

Figure 2 provides representative examples of flexible soil sensors reported in the literature.

## 6 Sensing Modalities

In soil sensing, a sensing modality refers to the underlying physical, chemical, or biological transduction mechanism by which environmental information is converted into a measurable signal. Soil sensing technologies are commonly classified into electrical, electrochemical, optical/spectroscopic, biological, and acoustic modalities [110]. While traditional rigid sensing platforms have explored all of these mechanisms, flexible soil sensing systems have predominantly focused on electrical and electrochemical approaches due to their compatibility with printed electronics, low‐power operation, and ease of integration onto mechanically compliant substrates. In contrast, optical, biological, and acoustic modalities are emerging as complementary strategies for non‐contact or highly selective soil characterization. Importantly, modality performance in flexible systems is governed by sensing physics, substrate mechanics, interfacial coupling, and long‐term stability under soil‐induced deformation.

### 6.1 Electrical Modality

Electrical sensing relies on changes in soil electrical properties, primarily resistance, capacitance, or impedance, induced by variations in moisture, ion concentration, and temperature. Its dominance in flexible soil sensing stems from simple device architectures, compatibility with scalable printing processes, and direct integration onto polymer substrates.

Resistive sensors transduce moisture‐dependent conductivity changes into resistance variation. Although attractive for low‐cost deployment, their performance is strongly influenced by salinity, temperature, and electrode polarization, leading to cross‐sensitivity and drift [111]. In flexible implementations, conformal contact reduces interfacial air gaps and improves repeatability; however, long‐term stability is limited by electrode degradation in ion‐rich environments. Capacitive sensors exploit the strong dielectric contrast between water and soil matrix components. Interdigitated electrode geometries fabricated on PET or PI substrates enable large‐area, flexible sensing patches that improve spatial averaging in heterogeneous soils [112]. Compared to resistive approaches, capacitive sensing reduces polarization effects but remains sensitive to salinity and temperature‐induced dielectric shifts. Chemoresistive sensors extend electrical transduction to gas or ion adsorption phenomena, enabling detection of soil‐emitted gases such as NH_3_, CO_2_, and CH_4_. CNT‐ and graphene‐based composites are commonly used due to their high surface area and mechanical compliance; however, exposure to moisture and particulate contamination can degrade sensing layers, necessitating encapsulation strategies [113]. Overall, electrical modalities are highly compatible with flexible platforms but suffer from limited selectivity in multiparameter soil environments where multiple physico‐chemical variables influence the same electrical response.

### 6.2 Electrochemical Modality

Electrochemical sensing translates ionic activity and redox processes into electrical signals, offering higher chemical specificity than purely electrical methods. These systems are widely used for monitoring pH, nutrients, heavy metals, and redox potential, and are typically classified into potentiometric, amperometric, voltammetric, impedance‐based, and conductimetric approaches [114, 115]. Potentiometric sensors measure ion activity through equilibrium potential differences governed by the Nernst equation. Flexible implementations using printed electrodes and ion‐selective membranes (ISMs) enable lightweight pH and nutrient sensing platforms. However, reference electrode instability and membrane fouling remain critical limitations in long‐term deployment [116]. Amperometric sensors rely on faradaic current generation from redox reactions, providing high sensitivity for trace analyte detection. Carbon‐based flexible electrodes (CNT, graphene, carbon inks) support high conductivity and mechanical durability, but are prone to biofouling and surface passivation under field conditions [117]. Voltammetric techniques (e.g., cyclic voltammetry (CV), square wave voltammetry (SWV)) enable multi‐analyte detection through redox fingerprinting, making them suitable for nutrient and heavy‐metal profiling. However, their requirement for controlled electrochemical interfaces limits direct in situ soil deployment in many cases [118, 119, 120]. Impedance‐based sensing bridges electrical and electrochemical domains by capturing frequency‐dependent soil response, enabling estimation of moisture, salinity, and organic content [121]. Interdigitated flexible architectures enhance spatial coverage, although interpretation remains model‐dependent due to soil heterogeneity. Conductimetric sensors provide bulk ionic conductivity measurements and are often used as salinity indicators. While simple and robust, they lack chemical specificity and are best used in multimodal sensor systems [114]. Collectively, electrochemical modalities provide strong chemical selectivity but require careful engineering of interfaces, encapsulation, and long‐term stability strategies in soil environments.

### 6.3 Optical and Spectroscopic Modalities

Optical sensing leverages light–matter interactions including absorption, reflection, scattering, and emission to infer soil properties. These approaches are attractive due to their non‐contact nature, reduced electrode degradation, and potential for multiplexed chemical analysis. Colorimetric sensors detect analytes through visually observable chemical reactions and are widely used for low‐cost detection of pH and nutrient species. Flexible implementations based on polymer films or paper substrates enable portable sensing patches; however, performance is strongly dependent on illumination conditions and soil solution uniformity [122]. Fluorescence‐based sensors offer higher sensitivity through molecular or nanomaterial emission responses. These systems enable selective detection of nutrients and pollutants but are limited by photobleaching, signal attenuation, and soil turbidity effects in real environments [123, 124]. Spectroscopic techniques, particularly near‐infrared (NIR) spectroscopy, provide non‐destructive, multi‐parameter soil characterization including moisture, organic matter, and mineral composition. While traditionally implemented as external instrumentation, ongoing miniaturization and integration with flexible platforms are enabling emerging in situ spectroscopic sensing concepts. However, these methods require complex calibration models and are sensitive to environmental scattering and soil heterogeneity [125, 126, 127]. Overall, optical modalities offer rich compositional information but face challenges in environmental robustness and field‐level integration.

### 6.4 Biological Modality

Biological sensors exploit biomolecular recognition elements such as enzymes, antibodies, deoxyribonucleic acid (DNA) probes, or microbial systems for highly selective detection of soil contaminants, pathogens, and nutrient biomarkers. Flexible platforms enable immobilization of biological receptors on polymer substrates through printing or functional coating techniques. Despite exceptional selectivity, biosensors remain limited by environmental fragility. Temperature fluctuations, microbial activity, and moisture variability in soil can degrade biological recognition elements, restricting long‐term field deployment. Current applications are primarily focused on short‐term diagnostics and targeted agricultural monitoring rather than continuous sensing [128].

### 6.5 Acoustic Modality

Acoustic sensing infers soil properties through mechanical wave propagation, where changes in velocity and attenuation correlate with density, compaction, moisture, and structural heterogeneity. Unlike electrical approaches, acoustic methods are inherently resistant to electrochemical corrosion and salinity interference [129]. Ultrasonic sensing has shown potential for soil compaction and moisture estimation; however, signal interpretation is complex due to scattering in heterogeneous media. Emerging flexible implementations based on polymer piezoelectric materials enable conformal acoustic sensing architectures for distributed soil characterization [130, 131].

### 6.6 Comparative Perspective for Flexible Soil Sensing

The effectiveness of each sensing modality in flexible platforms is fundamentally governed by the interplay between sensing physics, material selection, and soil–sensor interfacial mechanics. Electrical and impedance‐based modalities offer the highest compatibility with scalable flexible electronics and continuous monitoring but suffer from limited selectivity. Electrochemical sensors provide superior chemical specificity at the cost of increased interface complexity and stability requirements. Optical and spectroscopic approaches enable non‐contact or minimally invasive measurement with rich compositional information, but their performance is constrained by environmental variability and calibration complexity. Biological sensors provide unmatched selectivity but are limited by operational fragility in soil environments. Acoustic sensing offers a complementary, electrode‐free route for structural soil characterization but remains at an early stage of development. Across all modalities, sensor performance in flexible soil systems is ultimately determined by interfacial coupling efficiency, substrate mechanics, and long‐term environmental stability, highlighting the need for integrated multimodal designs rather than isolated sensing approaches.

## 7 Soil Sensor Materials

Soil sensors are designed with different sensing modes and material combinations depending on the nature of the parameter to be measured. Soil requires special attention in sensor design due to its high heterogeneity and variable physicochemical properties. The ability of sensors to perform reliable and repeatable measurements in the field depends not only on the performance of conductive and functional materials, but also on the properties of the substrates to which these materials are applied.

### 7.1 Substrates

Sensors operating in soil are subjected to various mechanical stresses such as bending, tension, compression, impact, torsion, and pressure [132, 133]. Therefore, an insufficiently flexible and durable substrate can cause the sensor to crack, deform, or lose signal. Soil exhibits dynamic variations in temperature and chemical properties, including pH, salinity, and mineral composition, which can significantly influence its physical, chemical, and biological behavior [134]. If the substrate is not resistant to fluctuations in temperature due to differences in weather conditions or chemical interactions, the conductive lines may be damaged, the substrate material may deform or begin to dissolve; this significantly reduces sensor performance. Soil is a moist environment, and the sensor is constantly in contact with water [31]. Specifically, in hydrophilic substrates, polymer chains form hydrogen bonds or dipole interactions with water molecules [135], which can cause the substrates to swell and change their mechanical properties, leading to instability in the sensor signal [136]. In addition, the surface energy, roughness, and chemical structure of the substrate directly affect the process of pressing or producing electrodes onto the substrate. Inadequate surface‐material compatibility leads to problems such as cracking, peeling, and increased contact resistance, negatively affecting the signal‐response accuracy of the sensor [137]. For sensors to operate reliably in soil environments for days, weeks, or months, the substrate used must demonstrate long‐term resistance to various environmental stresses such as UV light, microbial activity, abrasion, high humidity, and temperature fluctuations [138]. In fulfilling these performance requirements, the qualified and application‐specific selection of engineering polymers to be used as substrate materials plays a decisive role. Polymer substrates such as PI (Kapton), PET, polyethylene naphthalate (PEN), and PDMS are commonly preferred in flexible soil sensors (Table 1). The dominance of polymeric substrates is directly related to their flexibility, low elastic modulus and ability to withstand cyclic mechanical loading without fracture. This makes them particularly suitable for in situ soil applications where sensors are exposed to continuous deformation due to soil movement and external forces. Analysis of the data in Table 1 reveals that polymer‐based substrates stand out in flexible soil sensor designs due to their mechanical properties, chemical stability, and compatibility with fabrication techniques. Among these substrates, PET and Kapton are frequently used in studies, demonstrating a balance between cost‐effectiveness and high‐performance requirements. PET is commonly preferred for low‐cost and large area applications, while Kapton is more often used in chemically challenging and high temperature sensing environments. This trend highlights that substrate selection primarily depends on a balance between application area and ease of fabrication. Kapton is a highly preferred substrate, especially for ion‐selective electrodes and LIG‐based sensors, due to its high thermal resistance (up to 400°C) and chemical stability [139].

**TABLE 1:** Flexible soil sensors: Sensing modalities, conductive, functional, and substrate materials.

Categories	Sensing modality	Conductive material	Functional material	Substrate material	Detection target	Refs.
**Soil moisture** **sensor**	Capacitive	Cu film	—	PET film	—	[74]
	Capacitive	Silver nanoparticle (AgNP) ink	—	Paper	—	[140, 141]
	Capacitive	Carbon ink	—	PET	—	[142]
	Capacitive	Conductive ink	—	Nanocellulose‐coated biodegradable substrate	—	[143]
	Resistive	Graphene‐carbon ink	—	Paper	—	[144]
	Capacitive	AgNP ink	—	PET	—	[145]
	Capacitive	AgNP ink	—	PI film (Kapton)	—	[146]
	Capacitive	AgNP ink	—	Photo paper	—	[147]
**Soil temperature** **sensor**	Resistive	Silver (Ag) ink	—	PE‐based biodegradable mulch film	—	[148]
**Soil pH** **sensor**	Electrochemical square wave voltammetry	Ag/AgCI, carbon paste	Alizarin	Polypropylene (PP)(non‐tearable hydrophobic) paper	—	[87]
	Optical colorimetric	—	Bromocresol Green (BCG) and Bromocresol Purple (BCP)	Chromatography paper	—	[149]
	Electrochemical square wave voltammetry	Carbon ink, Ag/AgCI ink	Alizarin, salt membrane	PEN	—	[150]
	Electrochemical impedance	Graphene/carbon ink	Zinc oxide (ZnO) nanoparticles	Paper	—	[151]
**Soil nutrient**	Electrochemical potentiometric	Ag/AgCl	PVC‐based membranes containing nonactin and TDMAN	PI film (Kapton)	NH_4_^+^, NO_3_^−^	[80]
	Electrochemical potentiometric	AgNP ink, Ag/AgCI	NO_3_^−^ selective film	PI film (Kapton)	NO_3_^−^	[152]
	Electrochemical potentiometric	Gold (Au) ink, Ag/AgCI ink	NO_3_^−^ selective film	PET	NO_3_^−^	[115]
	Electrochemical	AgNP/iron oxide (Fe_2_O_3_) composite ink	Silver/iron(III) oxide (Ag^+^Fe_2_O_3_)/Nafion	Kapton film	NO_3_^−^	[153]
	Electrochemical amperometric	Carbon paste, silver	Multi‐walled carbon nanotubes (MWCNT)‐chitosan (CS) nanocomposite	Commercial flexible screen printed electrodes	Nitrite (NO_2_^−^)​	[154]
	Electrochemical cyclic voltammetry	Carbon, Ag/AgCl	Carbon black nanoparticles (CBNPs)	PDMS	PO_4_^3−^	[155]
	Electrochemical amperometric	Au, Ag/platinum (Pt), Ag/AgCl	ISM, PEDOT:PSS, potassium nitrate (KNO_3_) gel	Alumina (Al_2_O_3_) thick film	K^+^, NO_3_^−^	[81]
	Electrochemical impedimetric	AgNP ink, Au	Butylacrylate potassium ion selective membrane	PEN Q81 foil	K^+^	[156]
	Optical colorimetric	—	Reagents (sodium salicylate (C_7_H_5_NaO_3_), sodium nitroprusside, sodium dichloroisocyanurate)	Paper	NH_4_^+^	[157]
**Soil pollutant** **sensor**	Electrochemical differential pulse anodic stripping voltammetry	Carbon ink, Ag/AgCI ink	Nafion polymer and bismuth (Bi) film	Flexible PET	Pb^2+^and Cd^2+^heavy metals	[158]
	Electrochemical cyclic voltammetry (CV) and differential pulse voltammetry (DPV)	Carbon, Ag/AgCI, gold nanoparticles (AuNPs), polypyrrole (Au@PPy)	Pb^2+^ aptamer, Toluidine Blue, Complementary Strand DNA	Commercial screen printed carbon electrodes	Pb^2+^	[159]
	Electrochemical square wave anodic stripping voltammetry	Graphite‐based ink, Ag/AgCI ink	CBNP‐AuNPs	Flexible PET film	Hg^2^^+^	[160]
	Optical colorimetric, electrochemical	Carbon ink, graphite powder, MWCNTs	Bi, potassium ferricyanide, 1,10‐phenanthroline,Dimethylglyoxime (DMG), 1,5‐Diphenylcarbazide (1,5‐DPC), Bathocuproine	Filter paper	Colorimetric: iron (Fe), Cu, Nickel (Ni), Chromium (Cr) Electrochemical:Pb, Cd	[161]
	Optical colorimetric	‐ (no electronic electrode; relies on optical color change)	β‐galactosidase (B‐GAL)	Filter paper	Hg(II), Ag(I), Cu(II), Cd(II), Pb(II), Cr(VI), Ni(II)	[162]
**Soil** **multi‐modal sensors**	Chemiresistive, resistive	LIG	VO_x_‐doped LIG foam nanocomposite.	Pluronic F127‐resols hybrid film	NO_x_, temperature	[163]
	Electrochemical potantiometric, impedance	AgNP ink, SWCNTs dispensing ink,	PANI, Lityum Klorür/PVDF‐HFP	PET film	pH, humidity, temperature	[164]
	Electrochemical potantiometric, capacitance	AgNP ink, Cu	NO_3_^−^ ISM	PI	NO_3_^−^, humidity, temperature	[165]

Kapton reduces the risk of short circuits in humidity sensors thanks to its low water absorption rate and increases signal stability in electrochemical measurements [166]. PET is the most commonly used substrate in inkjet or screen‐printing techniques due to its flexibility, low cost, and ease of processing [167, 168]; its transparent structure also makes it suitable for optical and colorimetric sensor designs [169]. PEN is chemically very similar to PET and is usually produced by the polycondensation of ethylene glycol (EG) and naphthalene‐2,6‐dicarboxylic acid (NDA). Compared to PET, it has higher heat resistance (Tg ≈ 120°C), better chemical resistance, and superior dimensional stability [168]. PDMS, with its elastic and flexible structure, is preferred especially in bendable, conformal, and wearable sensor applications; performance can be optimized for moisture or ion‐selective measurements with hydrophilic or hydrophobic surface modifications [170]. Its low Young's modulus enables conformal contact with irregular soil surfaces, which is critical for maintaining stable sensor–soil interaction and consistent signal acquisition under dynamic conditions.

### 7.2 Conductive Materials

**FIGURE 3:**
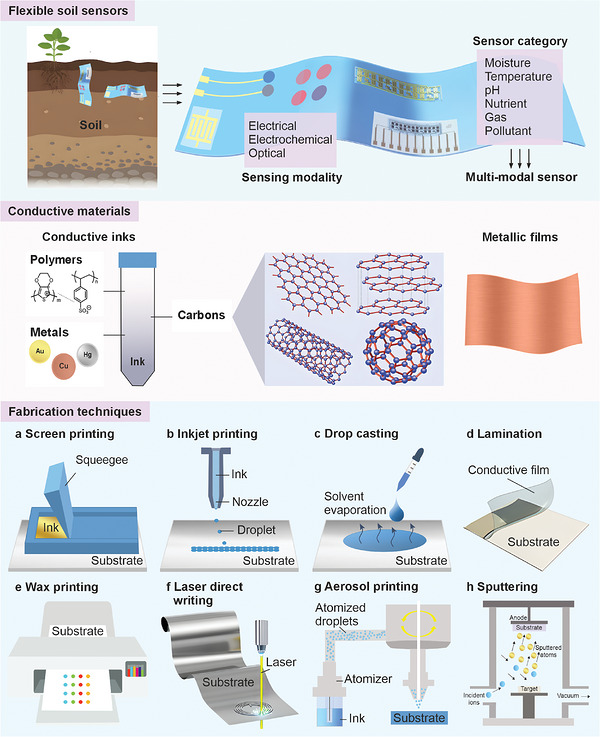
Overview of flexible soil sensors, conductive materials, and fabrication techniques. Flexible soil sensors for in situ monitoring of moisture, temperature, pH, nutrients, gases, and pollutants, using electrical, electrochemical, or optical modalities, with certain sensors combining multiple modalities. Conductive materials used include polymer‐ or metal‐based inks/films and carbon‐based nanomaterial inks. Fabrication techniques include screen printing, inkjet printing, drop casting, lamination, wax printing, laser direct writing, aerosol jet printing, and sputtering.

The conductive materials used in sensor fabricating vary depending on the type of sensor being produced, and this is a subject that requires detailed research. The conductive material used is directly related to signal quality. Ensuring stability in signal transmission is crucial for maintaining optimum sensor performance. Signal loss may occur in the heterogeneous structure of the soil. Therefore, the conductive material used in the electrodes of sensor must ensure that the signal travels without loss, minimizes soil‐induced noise, facilitates the easy transport of electrons, and ensures the repeatability of the measurement [171]. Table 1 and Figure 3 show the conductive materials used in flexible soil sensors. According to Table 1 and Figure 3, Ag‐based materials are the dominant choice, followed by carbon‐based alternatives for flexible soil sensors. This trend suggests that high electrical conductivity, signal stability, widespread use, and ease of adaptation to a wide variety of methods (different types of printing techniques, lamination or drop casting) are the dominant criteria in the selection of conductive materials. In addition, the increasing use of carbon‐based materials, particularly graphene and carbon inks, reflects a growing interest in cost reduction, flexibility, and improved surface activity. Furthermore, the distribution of materials reveals that silver‐based inks are predominantly used in capacitive and reference electrode systems, while carbon‐based materials are more frequently used in electrochemical sensing applications such as pH, nutrient, and pollutant detection. This indicates a functional differentiation in conductive material selection depending on the sensing mechanism and intended use. When Ag inks are examined more closely, they are the most commonly used type, categorized into two main groups based on their chemical composition and electrochemical function: AgNP ink and Ag/AgCl ink. AgNP ink primarily contains high‐purity metallic AgNPs to provide high electrical conductivity. They are particularly prominent as the basic electrode material in capacitive moisture sensors; Ag stability and low resistance make it ideal for sensitive applications that measure changes in water content through capacitance differences [140, 141, 145, 146, 147]. Ag/AgCI ink is a heterogeneous mixture containing metallic Ag and AgCI, a poorly soluble salt. In electrochemical sensing systems, this ink is used as a stable reference electrode based on a reversible half‐cell reaction. It provides a precise reference point against which variable signals from the active (working) electrode are compared to accurately determine the concentration of chemical species such as pH, nutrient ions, or contaminants [80, 81, 87, 115, 150, 152, 155, 158, 159, 160]. Although Ag‐based inks provide excellent conductivity, they are prone to microcrack formation under repeated bending, which may lead to signal instability in flexible applications. This limitation has driven the exploration of alternative materials. Carbon‐based inks, the second most preferred material after Ag, have been used primarily in electrochemical sensing (pH, nutrients, pollutant). These inks have different derivatives that offer application‐specific performance. Carbon ink serves as a general‐purpose electrochemical electrode, while graphene‐carbon ink offers high surface area properties thanks to the addition of graphene [142, 150, 151, 154, 155, 158, 159, 161]. Compared to metallic inks, carbon‐based materials exhibit superior mechanical compliance and strain tolerance, making them more suitable for flexible soil sensor applications. Their ability to maintain conductive networks under deformation is a key advantage in dynamic soil environments. Additionally, graphite‐based ink has been used in pollutant sensors (voltammetry) [160]. Unlike traditional ink usage, LIG is a process that converts a polymer film (usually PI) into graphene in situ by applying a laser beam. Its porous structure maximizes the sensor's surface area, enabling faster and more robust collection of chemical signals [80, 164]. In addition, the data suggest a shift toward advanced nanostructured materials such as laser‐induced graphene, which offer significantly higher surface area and improved signal responsiveness. This indicates an emerging trend in sensor design focusing on sensitivity enhancement and miniaturization. Also, Au has high conductivity, its high cost means it is generally only seen in sensors requiring very high sensitivity [156, 159].

### 7.3 Functional Materials

Functional materials determine the selectivity and sensitivity of sensors such as ISMs, redox dyes, nanoparticles, and biomaterials in chemical and electrochemical signal‐based pH, nutrient, and pollutant sensors. Since specific chemical selectivity is not required in soil moisture and temperature sensors, “functional materials” are not used. Unlike physical sensors, chemical soil sensors rely heavily on functional materials to achieve selectivity toward target analytes. Therefore, the choice of functional material is not arbitrary but directly linked to the sensing mechanism, required sensitivity and environmental conditions. A general trend observed in the literature is the use of simple indicator dyes in optical sensing, while nanomaterials and ISMs dominate electrochemical sensing approaches due to their higher sensitivity and specificity [114, 172, 173]. pH, nutrient, and pollutant measurement are a type of sensing that inherently requires chemical selectivity. For example, alizarin is used in pH sensors. It is an anthraquinone‐based dye and a classic pH indicator that changes color depending on the pH of the solution [87, 150]. Optical colorimetric indicators such as BCG and BCP also indicate pH changes through color changes [149]. These materials are widely preferred due to their simplicity, low cost, and ease of integration into flexible platforms. However, they are generally limited in terms of long term stability and quantitative accuracy. In electrochemical impedance‐based pH sensors, ZnO nanoparticles enable measurement by altering the surface charge depending on pH [151]. Compared to optical indicators, nanoparticle‐based materials provide higher sensitivity, faster response time, and better suitability for continuous monitoring, highlighting a clear balance between simplicity and performance. At the same time, nanostructured materials are particularly advantageous in flexible systems due to their ability to accommodate mechanical deformation without significant loss of functional performance.

In the measurement of nutrient ions, ISMs, nanocomposites, and catalytic surfaces are used. PVC‐based membranes and ionophores such as nonactin or tridodecylmethylammonium nitrate (TDMAN) provide high selectivity for specific ions such as NH_4_^+^ or NO_3_^−^ [80]. Nitrate‐selective films [115, 152], and butylacrylate‐based potassium‐selective membranes [156] also regulate ion transport in potentiometric or impedimetric measurements. The widespread use of ISMs indicates that selectivity remains the primary challenge in nutrient sensing. These materials enable targeted ion recognition. Nevertheless, their performance can be affected by interference from other ions present in complex soil environments. Electrochemical enhancing materials, such as Ag^+^Fe_2_O_3_/Nafion composites [153], MWCNT–CS nanocomposites [154], CBNPs [155], PEDOT:PSS, and KNO_3_ gel [81], increase the electrode surface area, accelerate ion transfer, and enhance the measurement signal. This reflects an important design strategy that combining selective layers with conductive nanostructures to simultaneously improve selectivity and signal strength. In optical nutrient sensors, reagents such as C_7_H_5_NaO_3_, sodium nitroprusside (SNP), and sodium dichloroisocyanurate (NaDCC) provide measurement through color change [157]. However, similar to pH indicators, these systems are generally more suitable for rapid and low‐cost detection rather than high precision continuous monitoring. In pollutant sensors, functional materials play a more critical role in terms of both selectivity and signal amplification. In the measurement of heavy metal ions, Bi films coated with Nafion polymer enable the pre‐enrichment of metal ions on the electrode surface [158]. DNA‐based aptamers and redox dyes such as Toluidine Blue are used for selective binding to specific metal ions and signal generation [140]. These biomolecular approaches offer extremely high selectivity, but, their practical application may be limited by stability and cost issues. CBNPs–AuNPs nanocomposites provide sensitivity in voltammetric measurements by offering high surface area and redox activity [160]. In optical pollutant sensors, complex‐forming reagents such as 1,10‐phenanthroline, dimethylglyoxime, 1,5‐diphenylcarbazide, and bathocuproine measure by changing color with metal ions [161]. Additionally, enzymes such as β‐GAL can indicate the presence of organic pollutants with an optical signal [162]. In general, the selection of functional materials used in flexible soil sensors should be made considering sensitivity, selectivity, and operational stability. According to studies in the literature, increasing importance is given to nanostructured and hybrid materials to overcome the limitations of traditional approaches.

## 8 Geotextiles as Emerging Substrates for Soil Sensing

Geotextiles are technical textile structures designed to perform basic engineering functions such as filtration, drainage, and stabilization [174], and in modern agriculture, they serve as a strategic substrate that optimizes water management in the root zone and prevents erosion [43]. Geotextiles, which are produced from synthetic polymers such as PP, PET, and polyethylene (PE), as well as from natural fibers, are configured in woven, knitted, nonwoven, knotted, geogrid, membrane, or composite forms [175, 176, 177]. The geometry, dimensions, and material compositions of these structures are optimized according to the mechanical strength, permeability, and environmental stability requirements of the application [178]. Accordingly, geotextiles are engineered to withstand field conditions such as UV radiation, moisture variation, temperature fluctuations, microbial activity, and chemical exposure, ensuring structural durability under harsh environmental conditions.

To establish a systematic design perspective for sensing applications, the intrinsic properties of geotextiles are directly related to key sensor performance requirements. High tensile strength, puncture resistance, and abrasion tolerance correspond to the mechanical robustness required for maintaining the integrity of embedded sensors under long‐term soil loading and deformation. The porous and fibrous architecture enables efficient mass transport, facilitating moisture and ion diffusion toward the sensing interface, which is essential for accurate environmental signal acquisition. In addition, low thermal conductivity contributes to thermal equilibration with the surrounding soil matrix, improving temperature sensing stability. Furthermore, resistance to UV radiation, moisture, and chemical exposure ensures long‐term preservation of both structural integrity and electrical signal stability in outdoor field conditions.

When considered as a substrate for soil sensing technologies, it offers certain advantages over traditional impermeable polymeric flexible thin films (PDMS, PET, or PI). Thanks to their high tensile strength, puncture resistance, and abrasion tolerance, geotextiles can maintain their structural integrity under long‐term mechanical loads in the soil. This property is particularly important for the durability of embedded temperature, humidity, and deformation sensors under field conditions. Traditional flexible substrates tend to form a physical barrier within the soil matrix due to their impermeable and hydrophobic nature, which can affect hydraulic and ionic continuity. In contrast, the porous, fibrous architecture of geotextiles provides a structure that facilitates the diffusion of moisture and target analytes to the sensor interface without restricting mass transfer. It is anticipated that this permeable nature can optimize detection sensitivity by minimizing the risk of “water pooling”—a phenomenon observed on non‐porous surfaces that negatively impacts data stability. Additionally, natural fiber geotextiles, preferred in line with a sustainability vision, minimize surface runoff caused by heavy rainfall due to their high water‐holding capacity. The controlled and steady release of stored water into the soil during dry periods creates an optimized environment (microclimate) for plant growth [43]. Geotextiles made from jute, coconut fiber, flax, and bio‐based polymers offer environmentally friendly alternatives for establishing sustainable sensor systems [179]. Additionally, the utilization of sheep's wool and feather waste in the production of biodegradable geotextiles has contributed to the stabilization of soil water regimes by releasing the stored water to plants over a long period and in a gradual manner, while also enriching the soil with nutrients during the degradation process and absorbing up to 45% of water from rainfall and evaporation [180]. Broda et al. (2018) found that wool‐based biodegradable geotextiles provide immediate protection against slope erosion in the initial stage, and that over time, as they biodegrade, they fertilize the soil through nitrogen‐rich compounds to promote plant growth, and ultimately, the developing vegetation takes the place of the geotextiles to ensure long‐term stabilization [181].

Existing studies on the use of geotextiles as sensor substrates have primarily focused on geotechnical applications such as strain, stress [182, 183], deformation monitoring [184], and moisture and temperature sensing [183, 185, 186], while research into the systematic use of geotextiles as active soil sensing platforms is still in its early stages. Fiber‐optic sensors have been preferred in these studies [187] and are generally integrated into geotextiles using embroidery technology. For example, Kuang et al. (2011) demonstrated that when plastic optical fiber (POF) sensors are integrated with geotextile structures, they maintain structural integrity and provide stable data transmission even under water pressure at a depth of 25 m and high strain conditions of up to 40% [188]. Similarly, within the scope of the POLYTECT project and the GeoDetect system, optical fiber sensors were successfully integrated into geotextile structures, enabling long‐term deformation and moisture monitoring in geotechnical applications [189, 190]. In another study, yarn‐based sensors composed of stainless steel fibers and polyacrylonitrile (PAN)‐based carbon fibers were integrated onto a geotextile substrate using an embroidery method, and moisture and deformation monitoring were performed through this structure [191]. These studies demonstrate that geotextiles can be used as functional platforms for sensor networks. However, in long‐term field applications, environmental factors such as humidity, water leakage, temperature fluctuations, UV radiation, wind, particle abrasion, and mechanical loads can affect the durability of geotextile systems. In particular, these conditions can lead to chemical degradation, photooxidation, hydrolysis, and loss of mechanical strength in synthetic polymer‐based geotextiles [192]. Additionally, UV radiation causes deterioration in the mechanical strength of geotextiles and geomembranes [193]. Therefore, depending on the application conditions, it is necessary to develop appropriate material and production strategies to protect smart geotextiles from environmental effects, such as UV stabilizer additives, coating/encapsulation techniques, multi‐layered composite structure designs, and interlayer architectures that isolate sensor integration.

## 9 Design Strategies and Fabrication Methods

The design strategies and manufacturing methods for sensors are quite diverse. Photolithography, thin‐film/thick‐film deposition, as well as printing technologies, have been used in the production of rigid soil sensors [165], while flexible soil sensors fabricated on conventional substrates such as PET, Kapton, and paper employ techniques such as screen printing, inkjet printing, drop casting, lamination, wax printing, laser direct writing, aerosol jet printing (AJP), and sputtering (Table 2 and Figure 3). As shown in Table 2, screen printing and inkjet printing are the most commonly used techniques.

**TABLE 2:** Fabrication processes used for different types of flexible soil sensors.

Categories	Fabrication process	Refs.
**Soil moisture sensor**	Lamination	[74]
	Inkjet printing	[140, 141, 142, 143, 144, 145, 146]
	Screen printing	[142, 143, 144]
**Soil temperature sensor**	Inkjet printing	[148]
**Soil pH sensor**	Screen printing	[87, 88, 89, 90, 91, 92, 93, 94, 95, 96, 97, 98, 99, 100, 101, 102, 103, 104, 105, 106, 107, 108, 109, 110, 111, 112, 113, 114, 115, 116, 117, 118, 119, 120, 121, 122, 123, 124, 125, 126, 127, 128, 129, 130, 131, 132, 133, 134, 135, 136, 137, 138, 139, 140, 141, 142, 143, 144, 145, 146, 147, 148, 149, 150, 151]
	Wax printing	[149]
**Soil nutrient sensor**	LIG	[80]
	Inkjet printing	[152]
	Screen printing and inkjet printing	[115]
	AJP	[153]
	Screen printing and drop casting	[81, 82, 83, 84, 85, 86, 87, 88, 89, 90, 91, 92, 93, 94, 95, 96, 97, 98, 99, 100, 101, 102, 103, 104, 105, 106, 107, 108, 109, 110, 111, 112, 113, 114, 115, 116, 117, 118, 119, 120, 121, 122, 123, 124, 125, 126, 127, 128, 129, 130, 131, 132, 133, 134, 135, 136, 137, 138, 139, 140, 141, 142, 143, 144, 145, 146, 147, 148, 149, 150, 151, 152, 153, 154]
	Screen printing	[155]
	Inkjet printing and sputtering	[156]
	Screen printing and dropping	[157]
**Soil pollutant sensor**	Screen printing	[158, 159, 160]
	Wax printing and screen printing	[161]
	Inkjet printing	[162]
**Soil multi‐modal sensor**	Laser direct writing	[163]
	Inkjet printing, sputtering	[164]
	Inkjet printing	[165]

Inkjet printing technology enables the creation of complex patterns at low cost and with minimal waste by depositing the material directly onto the targeted area. In this method, a thermal annealing step is typically required after printing to allow the ink to dry and achieve conductivity; this limits the choice of substrate materials. Therefore, substrates with high thermal stability, such as PI (Kapton), are preferred [146, 152, 153, 165]. In recent years, the traditional requirement for high‐temperature annealing has been reduced through innovative post‐printing processes such as plasma reduction, making it possible to apply inkjet printing to thermally sensitive substrates (PE, cellophane tape) [148]. Using this fabrication method, moisture [140, 141, 145, 146], temperature [148], nutrients [152], pollutants [162], and multimodal soil sensors [165] have been developed. For instance, inkjet‐printed sol–gel entrapped β‐GAL on Whatman no. 1 paper achieved detection limits as low as 0.001 ppm for Hg(II) with a 10 min response time and over two months of shelf life at 4°C [162]. One of the primary limitations of inkjet printing is nozzle clogging. the ink must exhibit appropriate viscosity and surface tension characteristics to ensure stable jet formation and reliable droplet deposition.

Screen printing is based on the principle of layering conductive inks onto substrate layers using stencils (screens). A separate stencil is used for each different color or pattern element, such as conductive traces or insulation layers. Production is carried out using manual screen printing presses or automated systems [147] and typically requires the sequential printing of layers followed by the application of appropriate curing steps after each layer [87, 142, 143, 150, 151, 155, 158, 159, 160]. Screen‐printing on a CNF‐infiltrated cardstock composite substrate has been successfully demonstrated for capacitive interdigitated soil moisture sensors, achieving a normalized capacitance sensitivity of 0.75 (large sensor) and 0.28 (small sensor) from 40% to 90% RH, a 9× capacitance increase from dry to 25% soil moisture, moisture absorption and desorption time constants of 6–8 and 4–5 min respectively, and repeatable response across multiple wetting‐drying cycles with less than 5.5% capacitance variation in dry soil between cycles [143]. However, the requirement for physical screens limits design flexibility, as even minor modifications necessitate fabrication of a new stencil, making this method less suitable for rapid prototyping.

Drop casting is a simple and rapid method for applying ISMs or nanocomposite materials to the active regions of sensors. This method requires the accurate application of solutions in volumes typically in the microliter range to the targeted area and usually involves drying at room temperature, ensuring compatibility with thermally sensitive substrates. For instance, drop casting of a KNO_3_ gel‐based ion‐permeable membrane onto screen‐printed thick‐film electrodes on an alumina substrate has been demonstrated for simultaneous amperometric K^+^ and NO_3_^−^ sensing in soil, achieving sensitivities of ∼0.6 µA/mM (R^2^ = 0.9775) and ∼2 µA/mM (R^2^ = 0.9708) respectively over a 0.1–10 mM range, with the fully integrated system successfully tracking fertilization and watering events in field conditions over multiple days of autonomous operation [81]. In contrast, low reproducibility, the “coffee‐ring” effect, and uncontrolled film morphology represent major limitations of this method. Therefore, it is not well‐suited for multisensor arrays or large‐area systems.

‐LIG method produces flexible graphene structures through a single‐step laser writing process, eliminating the need for complex conductive ink preparation, post‐printing annealing, or the use of metal catalysts. This process enables lithography‐free, chemical‐free production by converting hybridized carbon in the substrate into the graphene allotrope and can be performed at room temperature in air. Porous graphene is typically obtained in selective regions using fast‐pulsed infrared or low‐cost ultraviolet lasers, enabling the production of low‐cost, scalable sensors in the field. For instance, LIG electrodes fabricated on PI substrates using a 405 nm UV laser at an optimized 20 ms pulse width and subsequently functionalized with PVC‐based ionophore membranes via drop casting have been demonstrated for solid‐contact ion‐selective electrochemical sensing of soil nitrogen, achieving near‐Nernstian sensitivities of 51.7 ± 7.8 mV/dec for NH_4_^+^ and −54.8 ± 2.5 mV/dec for NO_3_^−^, detection limits of 28.2 ± 25.0 µM and 20.6 ± 14.8 µM respectively, a linear range of 10^−^^5^–10^−^^1^ M, long‐term drift as low as 0.93 mV/h, and average signal recovery of 96% and 95% in complex soil slurry matrices [80]. However, the primary limitation of the LIG method is its compatibility only with carbon‐containing substrates, as well as its relatively poor resistance to mechanical wear. In soil environments, long‐term use may lead to surface abrasion, resulting in performance degradation in sensing performance.

Wax printing is a widely used production technique for creating hydrophobic barriers, particularly in paper‐based analytical devices (PADs). In this method, desired patterns are printed onto paper using wax printers, and then a thermal process is applied to ensure the wax penetrates the paper fibers; this process is typically performed on hot plates. The heating process allows the wax to impregnate the paper, creating hydrophobic barriers and hydrophilic channels between them. These structures are used in colorimetric analyses to direct the controlled flow of the solution through the device and isolate the liquid distribution [149, 161]. For instance, wax‐printed Whatman grade 1 filter paper has been demonstrated in a three‐dimensional multilayer PAD integrating simultaneous colorimetric detection of Fe, Ni, Cu, and Cr and electrochemical detection of Pb and Cd from a single sample, achieving colorimetric detection limits of 0.12 µg for Cr, 0.75 µg for Fe, Cu, and Ni, and electrochemical detection limits of 0.25 ng (1 µg/L) for both Pb and Cd via square‐wave anodic stripping voltammetry, with measured values statistically indistinguishable from certified reference values in real particulate matter samples [161]. However, wax printing generally provides lower resolution.

AJP is an advanced printing technology that enables the creation of high‐resolution and complex patterns by spraying functional inks onto the target surface in the form of very fine aerosol droplets. In this method, the ink is atomized by a gas stream and directed onto the target substrate, forming layers on the surface with the desired thickness and accuracy. AJP offers higher resolution and three‐dimensional patterning capabilities compared to inkjet and screen printing. It can also be applied to flexible, curved, or irregular surfaces. For instance, AJP of AgNP ink onto a flexible Kapton substrate using a 200 µm nozzle has been demonstrated for a dual‐functional soil sensor combining an electrochemical NO_3_^−^ sensor and a capacitive moisture sensor on a single platform, with the NO_3_^−^ sensor achieving a linear detection range of 5–400 mg/L directly in saturated silt loam soil and the moisture sensor resolving volumetric water content from 0.1 to 0.25 m^3^/m^3^ in sandy soil [153]. However, high equipment cost and limited scalability represent significant constraints.

Sputtering is a physical vapor deposition (PVD) technique that enables the deposition of thin film layers onto a substrate from high‐purity materials. In this method, the target material is bombarded with ions in a plasma environment, releasing atoms or molecules that are directed toward the substrate surface. Sputtering enables the creation of conductive, semiconductive, or dielectric films with high thickness control and homogeneity [156, 164]. For instance, sputtering of 100 nm Au through an 80 µm thick wafer dicing tape mask onto a flexible PEN substrate has been demonstrated for reference‐electrode‐free impedimetric K^+^ sensing via electrochemical impedance spectroscopy, achieving a sensitivity of 4.553 MΩ/(mmol/L) at 100 mHz, an accuracy of 1.8 mmol/L over a 0.1–25 mmol/L range, a signal drift below 0.5%/h over 2 h, a response time below 40 s, and a batch‐to‐batch reproducibility error of less than 6.86% across four independently fabricated sensors [156]. However, the requirement for vacuum‐based systems and the low production rate constitute significant disadvantages for field‐scale manufacturing.

Lamination is employed not only to protect sensors and ensure their reliable long‐term operation in harsh soil conditions but also as a means of integrating sensor components. For instance, a Cu film patterned in an interdigital configuration is laminated with a PET substrate, creating the functional sensor structure while simultaneously providing mechanical and environmental protection [74].

A comparative summary is presented in Table 3 to provide an evaluation of the fundamental differences among fabrication methods and to guide researchers in selecting appropriate techniques during the manufacturing process. Inkjet printing and screen printing are among the most widely used techniques owing to their high compatibility with flexible substrates (e.g., PET, Kapton, textiles) and their suitability for scalable production. These two methods stand out as the most practical approaches for both academic research and industrial applications in the large‐scale fabrication of soil sensors.

**TABLE 3:** Comparison of fabrication methods used in flexible soil sensors.

Fabrication process	Resolution	Scalability	Compatible substrate	Post‐processing	Cost
Inkjet printing	High	Medium	PI, PET, paper, treated textiles	Thermal sintering	Low–Medium
Screen printing	Medium	High	Wide range of substrates	Layer‐by‐layer curing	Low
Drop casting	Low	Low	Wide range of substrates	Room temperature drying	Very low
LIG	Medium–High	Medium‐High	Carbon‐containing polymers (PI, wood, lignin paper)	Not required	Low
Wax printing	Low–Medium	Medium	Paper, cellulose‐based substrates	Thermal treatment (hot plate)	Very low
AJP	High	Low	Flexible, curved, and irregular substrates	Substrate‐dependent	High
Sputtering	High	Low	Rigid and flexible flat substrates	Not required	Very high
Lamination	Depends on pre‐pattern	High	Polymer films, textiles, multilayer composites	Not required	Low

The selection of the fabrication method depends not only on the printing technique itself but also on the rheological properties of the functional inks or pastes used. For instance, inks employed in inkjet printing must exhibit low viscosity and appropriate particle size to ensure reliable nozzle ejection. Metal oxides with large particle sizes or highly viscous active materials may induce nozzle clogging in inkjet systems; therefore, in such cases, screen printing or drop casting methods, which are more compatible with high‐viscosity formulations, are preferred. Similarly, the choice of fabrication method becomes critical in sensor applications requiring high resolution and precise pattern definition. In sensors with small active areas where highly accurate signal acquisition is required, drop casting may be insufficient due to the risk of uncontrolled spreading; therefore, higher‐resolution techniques such as AJP or sputtering are preferred.

Substrate thermal stability is another key parameter in method selection. If high‐temperature curing or sintering is required during sensor fabrication, low‐melting‐point substrates such as PET or paper may be unsuitable due to thermal limitations. In such cases, either alternative approaches such as LIG, which enables localized thermal processing, should be employed, or substrates with higher thermal stability should be used. Overall, the fabrication method should be optimized not only in terms of cost and resolution but also with respect to material compatibility, thermal processing requirements, and the performance expectations of the target application.

Despite these advances, the adaptation of these sensor fabrication methods, including inkjet printing, screen printing, AJP etc. to geotextile substrates has not yet been investigated, and critical challenges such as ink penetration into fiber interstices, adhesion loss under mechanical deformation, and compatibility with surface pre‐treatment and encapsulation protocols remain unaddressed; therefore, future research should prioritize the systematic optimization of both fabrication processes and material formulations specifically for geotextile integration in soil sensing applications.

## 10 Soil Parameter Measurement and Analysis Methods

Reliable and comparable measurements obtained with flexible soil sensors depend not only on suitable materials and device architectures, but also on calibration and validation against recognized reference methods. Because soil is a heterogeneous medium with strongly varying physicochemical properties, international standards provide the analytical basis for defining the target parameter, establishing calibration procedures, and interpreting sensor outputs under both laboratory and field conditions. As outlined in Table 4, standards such as DIN ISO 10390 for pH, ISO 11465 and ASTM D2216 for moisture/water content, DIN ISO 11265 for electrical conductivity, ISO 11260 and ISO 14255 for nutrient extraction, ISO 16072 and ISO 10381–6 for gases, and ISO 11466, ISO 13856‐2, and ASTM D3559 for pollutants should therefore be considered reference methodologies for calibration and validation rather than alternatives to the sensors themselves.

**TABLE 4:** International standards and reference methods for soil parameter analysis used in sensor calibration and validation.

Soil parameter	Standard reference	Description / Method
**Organic Carbon (SOC)**	DIN ISO 10694:1996 ISO 14235:1998	Determination of organic and total carbon by dry combustion (elemental analysis) or sulfochromic oxidation method. Used as a reference for validating soil organic carbon sensors
**pH**	DIN ISO 10390:2005	Determination of soil pH in CaCl_2_, KCl, or water suspension using a glass electrode. Standard method for calibration of soil pH sensors.
**Moisture / Water Content**	ISO 11465:1993 ASTM D2216	Gravimetric method based on mass difference before and after drying. Serves as reference for resistive or capacitive soil moisture sensors.
**EC**	DIN ISO 11265:1997	Determination of specific electrical conductivity of soil solution. Used to calibrate EC or salinity sensors.
**Nutrient Elements (N, P, K, Mg)**	ISO 11260:2018 ISO 14255:1998	Extraction of cations and anions (e.g., NO_3_^−^, PO_4_^3−^, NH_4_^+^) using standardized chemical solutions such as Ca‐acetate‐lactate (CAL). Provides reference values for ion‐selective nutrient sensors.
**Gases (O_2_, CO_2_, CH_4_, N_2_O, NH_3_)**	ISO 16072:2002 ISO 10381–6:2009	Standard laboratory and sampling methods for determining microbial respiration and soil gas composition. Used to validate gas sensor performance.
**Pollutants (Heavy Metals, Pesticides)**	ISO 11466:1995 ISO 13856‐2:2003 ASTM D3559	Determination of trace elements soluble in aqua regia and organic contaminants via GC‐MS. Provides reference data for chemical pollutant sensors.
**Texture and Physical Properties**	ISO 11277:2020	Determination of particle size distribution (sand, silt, clay) in mineral soils. Used to assess the influence of soil type on sensor performance.

Accordingly, the analytical assessment of flexible sensors should not be based solely on isolated performance indicators such as sensitivity, response time, or accuracy. Such values become meaningful only when interpreted together with the reference matrix, calibration route, and validation strategy. This is particularly important for flexible devices, since their analytical behavior depends not only on the sensing principle but also on how effectively they conform to the soil interface. As discussed in earlier sections, flexible structures can reduce air gaps and improve interfacial contact with irregular soil surfaces, thereby improving signal stability and enabling more representative in situ measurements.

Among soil parameters, moisture measurement provides a clear example of the difference between a reference analytical method and a sensor‐derived estimate. Standard methods such as ISO 11465 and ASTM D2216 determine moisture content through gravimetric mass loss before and after drying, whereas flexible resistive and capacitive sensors infer water content indirectly from changes in electrical properties. For this reason, sensor signals must be calibrated either against known moisture levels or against a trusted reference instrument. As summarized in Table 1, flexible moisture sensors have been implemented on substrates including paper, PET, biodegradable nanocellulose‐based materials, and PI films. In resistive flexible sensors, humidity‐responsive behavior has frequently been characterized first under controlled relative‐humidity conditions and then discussed in relation to agricultural use. In the graphene–carbon ink paper‐based sensor, measurements over the range of 25%–91.7% relative humidity (RH) produced a resistance variation of approximately 12.4 Ω/%RH, indicating high humidity sensitivity. The same study reported a response time of approximately 4 s and a recovery time of 6 s, together with stability extending beyond 4 months and repeatability over more than 100 humidity cycles [144]. From the perspective of flexible electronics, these findings are significant because they show that the sensing response remains stable during repeated operation and extended use. The device was also mechanically tested under bending radii of up to 20 mm and twisting angles of up to 90°, with only <6% change in base resistance, indicating that flexibility can preserve sensor functionality under deformation. However, these results should be interpreted as evidence of device robustness and humidity‐response capability rather than as direct proof of standardized soil‐moisture validation. By comparison, capacitive flexible sensors are more directly related to soil‐moisture assessment because they detect variations in dielectric properties associated with water content in the soil. In the biodegradable cellulose‐based sensor study cited as [143], moisture‐responsive capacitive behavior was examined on a nanocellulose‐coated substrate under both humidity and soil‐moisture conditions, showing that substrate choice can influence not only sensitivity but also signal interpretation. Since this type of platform is hygroscopic, analytical evaluation must account for both the capacitance change caused by the target parameter and the contribution of the substrate itself to the measured response. This consideration is particularly important for flexible and biodegradable systems, where the same material that improves conformability may also alter moisture uptake and response dynamics.

A more direct example of soil‐moisture sensing is provided by the printed interdigitated electrode sensor fabricated on flexible PI and reported in [146]. In that study, capacitive sensors were evaluated in soil over a volumetric water content (VWC) range of 0.05–0.25 m^3^/m^3^, which is analytically more consistent with standard moisture assessment because the electrical response was correlated with a defined soil‐water‐content interval rather than only with ambient humidity. This study is also important from the standpoint of flexibility, as the authors explicitly selected a flexible substrate to enable conformability on nonplanar sensing supports such as rods inserted into soil. In this respect, flexibility was not simply a mechanical convenience, but a design feature expected to improve measurement quality under practical deployment conditions. Taken together, these studies indicate that the measurement and interpretation of flexible soil‐moisture sensors should be addressed through a layered framework. First, the target variable should be defined through a reference approach such as gravimetric moisture determination. Second, the sensor output should be related to a known physical quantity such as RH or VWC. Third, the role of flexibility in contact quality, deformation tolerance, and long‐term signal preservation should be explicitly discussed. In this sense, flexibility is not merely a structural characteristic; it can directly affect analytical performance by improving conformity to heterogeneous soil surfaces, reducing interfacial artifacts, and maintaining functional stability under bending or twisting.

A comparable analytical perspective can also be extended to soil temperature sensing, although here the emphasis is less on chemical reference standards and more on measurement configuration, environmental robustness, and signal stability under realistic operating conditions. In this context, validation depends on whether the sensor can preserve electrical continuity, thermal sensitivity, and structural integrity while exposed to repeated deformation and fluctuating outdoor conditions.

Flexible temperature sensors developed for soil monitoring were subjected to bending tests at different radii in order to evaluate electrical continuity and mechanical strength. Additional thermal tests, including heating and freezing of the soil, demonstrated that the proposed system remained stable under changing temperature conditions [194]. In another study, a dedicated oven was constructed to determine the temperature sensitivity of sensors designed for soil‐temperature monitoring. The resistance of the thermistor was measured using a voltage and current source, after which the sensor was deployed outdoors in a garden environment. Hourly resistance measurements obtained with a multimeter confirmed that the proposed device was suitable for outdoor operation [148]. Taken together, these studies indicate that, for flexible soil‐temperature sensors, analytical reliability should be assessed not only in terms of thermal response, but also with respect to mechanical endurance and environmental stability under practical deployment conditions.

A similar reasoning applies to pH, EC, and nutrient sensing. For pH, DIN ISO 10390 provides the glass‐electrode‐based reference method in soil suspensions, and flexible pH sensors should therefore be interpreted in relation to whether calibration was carried out in buffer solutions, soil extracts, or direct soil contact. For electrical conductivity, DIN ISO 11265 defines the reference determination of soil‐solution conductivity, while nutrient sensing is generally interpreted relative to standardized extraction methods such as ISO 11260 and ISO 14255. In all of these cases, reported sensitivity values should not be considered in isolation, since matrix effects, competing ions, moisture level, and interfacial soil contact can substantially alter the response. The analytical relevance of each reported value therefore depends on the calibration medium and validation protocol used. For example, electrochemical pH sensors modified with graphene–carbon and ZnO nanoparticles exhibited an almost three‐fold impedance change over the pH range of 2–9 and a linear response with a sensitivity of 5.27 kΩ/pH in the pH range of 2–8; however, performance deterioration after three cycles indicated that the device was intended for single‐use operation [151]. Alizarin‐modified voltametric sensors tested by square wave voltammetry showed high sensitivity in buffer solutions, while the relative error remained limited to 4.9% in real soil samples [87]. In simplified two‐electrode systems, the use of Nafion and salt membranes improved stability, yielding a sensitivity of 6.38 mV/pH and a pH resolution of 0.28 in real soil and hydroponic environments [150]. Likewise, paper‐based colorimetric sensors combined with a smartphone‐based artificial intelligence platform achieved 97% accuracy in field classification of low, medium, and high pH levels through image‐based analysis [149]. These examples demonstrate that pH sensor performance must always be interpreted in relation to the testing matrix and the intended application environment.

For nutrient sensors, validation studies generally focus on four main aspects in order to demonstrate practical applicability. First, basic analytical performance is assessed through the linear range, sensitivity, and detection limit of the sensor, for example −54 mV/dec for NO_3_^−^ [115], 4.553 MΩ/mmol/L for K^+^ [156], and a detection limit of 1.2 µM for PO_4_^3−^ [155]. Second, chemical selectivity is examined to evaluate interference effects within the soil matrix; this includes, for instance, the low cross‐sensitivity of K^+^ sensors to sodium [156] and the high analytical recovery of the PO_4_^3−^ microfluidic platform, which exceeded 91% [155]. Third, mechanical and electrical stability are investigated to verify durability under bending and to assess signal drift, as demonstrated by flexible LIG sensors [80] and by an impedimetric design that avoided the need for a reference electrode and thereby improved long‐term stability [156]. Fourth, field application tests are conducted in real soil types, soil solutions, and soil columns to demonstrate practical monitoring capability [80]. In particular, multi‐sensor systems have been shown to measure N, K, pH, and moisture simultaneously with high accuracy, continuously and in situ [81]. Framing nutrient sensor performance in this way is more informative than simply listing isolated numerical values, because it clarifies how analytical capability is established from laboratory characterization through to realistic deployment.

For pollutant sensing, matrix specificity is especially important. Standards such as ISO 11466, ISO 13856‐2, and ASTM D3559 are relevant because they provide reference procedures for determining trace elements and organic contaminants in soil‐related analysis. Accordingly, only studies performed in soil or soil‐relevant matrices should be used as primary evidence of soil pollutant sensing performance. In this context, flexible pollutant sensors have been evaluated using hybrid strategies that combine highly sensitive electrochemical methods, such as differential pulse stripping voltammetry (DPASV) and square wave stripping voltammetry (SWASV), with colorimetric detection approaches based on enzyme inhibition. Within soil‐relevant matrices, screen‐printed electrodes (SPEs) modified with Nafion/Bi and operated according to the DPASV principle were successfully used to analyse Pb^2^^+^ and Cd^2^^+^ ions in soil samples [158]. An Au/PPycomposite aptasensor was also able to detect Pb^2^^+^ ions in soil with a very low detection limit of 0.6 ppb at S/N = 3 [159]. In addition, paper‐based colorimetric sensors using the β‐GAL enzyme detected Hg(II) ions down to 0.001 ppm (1 ppb) within 10 min, and the obtained results were consistent with conventional methods [161]. By contrast, studies performed in non‐soil media, such as river water or air particulate matter, may still demonstrate the sensitivity of a given sensing platform, but they should not be treated as direct validation of soil pollutant sensing. Maintaining this distinction is essential for analytical rigor and for ensuring that flexible sensor performance is interpreted within the appropriate environmental context.

Overall, the assessment of soil parameter measurement with flexible sensors should integrate four complementary components: the standardized definition of the target parameter, the underlying electrical or electrochemical sensing mechanism, the calibration and validation pathway, and the effect of flexibility on the sensor–soil interface. When these dimensions are considered together, reported performance values become analytically meaningful rather than remaining isolated literature metrics. Such an integrated perspective is particularly important for future geotextile‐based sensor systems, where long‐term soil contact, mechanical adaptability, and multi‐parameter sensing are expected to play central roles.

## 11 Energy Management, Wireless Communication Technologies, and IoT Integration

Power sources are another important element for sensors. Rigid sensors can be powered by batteries or electricity. Lithium–thionyl chloride batteries (Li‐SOCl_2_) have been used in rigid sensors. In agriculture, wired power sources are disadvantageous due to immobility, installation difficulties, maintenance costs, and the risk of environmental damage. Power supply issues, such as cable breaks due to agricultural machinery or animals, can occur. Furthermore, to reduce the carbon footprint, wireless connections are generally preferred in agricultural sensors, using solutions such as solar panels, batteries, or energy harvesting (triboelectric, piezoelectric, thermal, etc.) [195].

Most traditional wireless sensing devices used in agricultural fields are not sustainable [39, 196, 197]. These systems typically require additional energy consumption from batteries and need to be manually replaced frequently when the energy runs out. Although this increases precision and efficiency in agricultural production, it leads to an energy consumption model that further increases carbon emissions. Therefore, self‐powered wireless sensing systems are a more effective solution in smart agriculture. Environmental noise (acoustic energy), electromagnetic energy (RF) from wireless communication signals, or microbial energy produced by soil and environmental microorganisms can be considered alternative energy sources in agricultural systems [195].

Soil sensors alone cannot fulfil their promise without connectivity and analytics, their data remain isolated. The IoT provides the framework that links distributed sensors, enabling real‐time monitoring, data exchange, and automated control across farms. By establishing such communication networks, IoT transforms stand‐alone sensors into connected, intelligent systems that support precision agriculture. For instance, ion‐selective NO_3_^−^ and moisture sensors are integrated through a long range wide area network (LoRaWAN) based node for reliable long‐range transmission [198].

Standard IoT systems are typically structured in three layers, however in some systems the systems are expanded to five layers by adding object and user layers to capture field‐level sensing and human–system interaction [199]. This structure ensures seamless communication from soil sensors through gateways to cloud or edge platforms where data are analyzed and visualized for decision support.

IoT communication choices depend on distance and energy constraints. Short‐range networks such as ZigBee and Bluetooth Low Energy (BLE) are common in greenhouses due to their low power use and simple mesh configuration. Long‐range systems, including LoRaWAN and narrowband Internet of Things (NB‐IoT), dominate open‐field monitoring [199, 200]. LoRaWAN is preferred because it offers kilometer‐scale coverage, very low energy consumption, and license‐free deployment, which makes it ideal for isolated farms. However, researchers also reported limited data rate, duty‐cycle restrictions, and delayed downlinks in dense networks [201]. NB‐IoT provides higher reliability via cellular infrastructure but depends on operator coverage and subscription costs.

Recent studies integrate multiple parameters like NPK, pH, EC, moisture, and temperature within a single node for holistic soil assessment [198, 201]. These nodes aggregate data via RS‐485 or I^2^C communication and transmit through LoRaWAN or ZigBee gateways. Edge processing, reduces latency and power consumption by analyzing data locally before cloud upload [200]. Cloud servers then perform higher‐level fusion, generating indices such as the Soil Health Index (SHI), which supports fertilizer management and nutrient‐optimization strategies in smart‐farming applications [201].

Although IoT integration has advanced soil monitoring, flexible and textile‐based sensors are rarely networked in current literature. Future research should focus on developing textile multi‐sensor networks capable of detecting NPK/pH/EC/moisture and communicating via LoRaWAN or NB‐IoT. Combining such devices with AI‐based analytics would enable truly autonomous, self‐sustaining monitoring systems for next‐generation smart agriculture [23].

**FIGURE 4:**
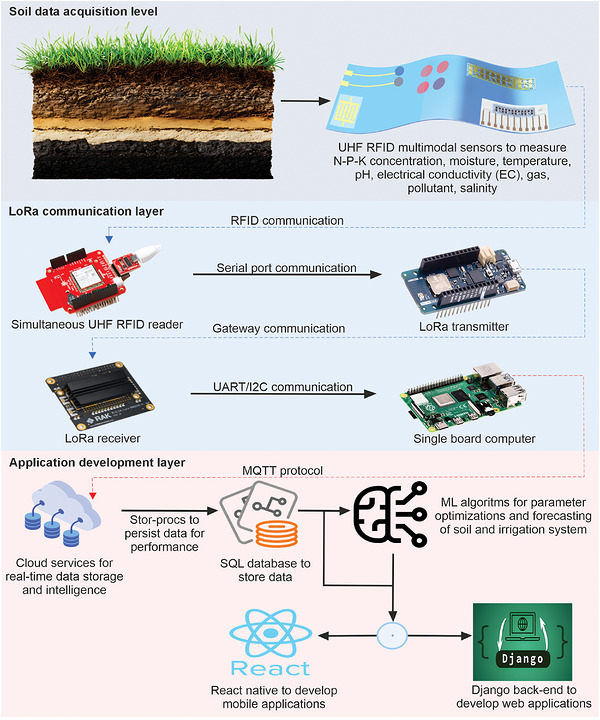
System architecture for smart agriculture utilizing low‐power wide‐area network (LoRa) and UHF RFID technology via geotextile sensor network. The architecture is composed of three main layers: the soil data acquisition level (UHF RFID passive sensors), the LoRa communication layer (featuring a simultaneous UHF RFID reader, LoRa transmitter/receiver, and single board computer for data transmission), and the application development layer.

For instance, the proposed system outlined in Figure 4 can serve as a viable IoT‐based data acquisition solution for soil monitoring. This data acquisition system proposes a multilayer IoT‐based architecture for high‐resolution, real‐time soil monitoring, integrating heterogeneous sensing modalities with long‐range wireless communication. In the sensing layer, passive ultra‐high frequency radio frequency identification (UHF RFID) –enabled devices acquire key physicochemical soil attributes, including N‐P‐K concentration, moisture, and pH, while EC sensors quantify ionic activity and salinity levels, and resistance‐based probes capture volumetric water content with high sensitivity [202, 203]. The acquired measurements are aggregated through an RFID reader and relayed to a LoRa transceiver via serial communication. Leveraging the low‐power, wide‐area characteristics of LoRa, the system ensures robust data transmission to a receiver node, where a single‐board computer performs gateway operations using UART/I^2^C interfaces [204]. Data is subsequently streamed to a cloud backend through the Message Queuing Telemetry Transport (MQTT) protocol, enabling scalable, real‐time data ingestion, storage, and analytical processing [205]. At the application layer, machine learning models are deployed to infer irrigation requirements, optimize nutrient management, and evaluate soil productivity, whereas Django‐based web interfaces and React Native mobile applications provide end‐users with advanced visualization and decision‐support capabilities [206]. Overall, the proposed architecture offers a modular, energy‐efficient, and analytically enhanced framework that aligns with contemporary precision agriculture paradigms and contributes to the development of autonomous, data‐driven agronomic management systems via geotextile sensor network structures.

## 12 Challenges and Future Directions

During the development of flexible soil sensors, a number of environmental and technical challenges are encountered. The reliable and long‐term performance of sensors under different soil types and variable environmental conditions (temperature, humidity, salinity) remains a major limitation. In addition, the correct installation of sensors is also critical; misplaced sensors can produce misleading data and lead to incorrect irrigation or management decisions.

Most soil sensors are characterized in laboratory settings using buffer solutions or controlled soil samples, with tests designed to assess sensitivity, linearity, and short‐term repeatability. However, the long‐term field performance of sensors, their responses to different soil types and variable environmental conditions, their dynamic behavior, and their mechanical durability are often insufficiently investigated.

The materials and production techniques used in sensor manufacturing play a critical role in both performance and sustainability. Polymeric and biodegradable materials provide flexibility to sensors while ensuring environmental compatibility; however, properties such as high‐temperature resistance, chemical stability, and long‐term mechanical strength remain limiting factors, particularly in outdoor applications. Nanomaterials, conductive polymers, and print‐based manufacturing methods can increase the sensitivity and multi‐parameter measurement capabilities of sensors; however, cost, manufacturing reproducibility, and suitability for large‐scale applications remain important issues to be resolved. Furthermore, flexible substrates and coatings should be complemented by advanced surface engineering and encapsulation techniques to enhance the sensors' resistance to moisture, salinity, and mechanical effects.

Future studies aim to overcome existing technical and environmental challenges. In particular, functionalizing geotextiles with flexible or stretchable conductive materials to develop environmentally friendly and cost‐effective agricultural multimodal sensing technologies represents a promising research direction. Furthermore, the integration of sensor data with smart farming systems can be achieved through autonomous decision‐making mechanisms, high‐density sensor networks, and energy‐efficient modules. Artificial intelligence and machine learning‐based data analysis methods will contribute to the technical and environmental sustainability and cost‐effectiveness of future sensor design and production by optimizing the processing, calibration, and multi‐parameter monitoring of field data.

## 13 Conclusion

This review highlights the evolution of soil sensors used in agriculture from rigid structures to flexible and geotextile‐based systems, providing a comprehensive assessment of technological developments in this field. Rather than being examined under separate headings such as moisture, temperature, pH, nutrient, and pollutant sensors according to traditional classification methods, the sensors are addressed holistically within the framework of fundamental themes such as materials science, production technologies, testing methods, energy management and IoT integration. This common framework enables direct comparison between different sensor types and highlights existing similarities, shortcomings, and research gaps in the literature more clearly.

## Funding

European Commission under HORIZON‐TMA‐MSCA‐SE Action project “IoT Supported Electronic Geotextiles for Sustainable and Smart Precision Agriculture (I‐TEXGEO)” (grant number: 101235387) and Scientific and Technological Research Council of Türkiye (TÜBİTAK) under the 2247‐A National Outstanding Researchers Program “Development of an Artificial Intelligence–Assisted, IoT‐ and E‐Textile–Based Sensor Technology for Soil Analysis, Monitoring, and Smart Fertilization in Agricultural Applications” (grant number: 123C518).

## Conflicts of Interest

The authors declare no conflicts of interest.

## Data Availability

Data sharing not applicable to this article as no datasets were generated or analysed during the current study.
